# Mouse Norovirus Infection Arrests Host Cell Translation Uncoupled from the Stress Granule-PKR-eIF2α Axis

**DOI:** 10.1128/mBio.00960-19

**Published:** 2019-06-18

**Authors:** Svenja Fritzlar, Turgut E. Aktepe, Yi-Wei Chao, Nathan D. Kenney, Michael R. McAllaster, Craig B. Wilen, Peter A. White, Jason M. Mackenzie

**Affiliations:** a Department of Microbiology and Immunology, University of Melbourne at the Peter Doherty Institute for Infection and Immunity, Melbourne, Victoria, Australia; b Department of Pathology and Immunology, Washington University School of Medicine, St. Louis, Missouri, USA; c Departments of Laboratory Medicine and Immunobiology, Yale School of Medicine, New Haven, Connecticut, USA; d School of Biotechnology and Biomolecular Sciences, The University of New South Wales, Sydney, New South Wales, Australia; Indiana University Bloomington

**Keywords:** integrated stress response, mouse norovirus, protein kinase R, protein translation, stress granules, eIF2α

## Abstract

The integrated stress response (ISR) is a cellular response system activated upon different types of stresses, including viral infection, to restore cellular homeostasis. However, many viruses manipulate this response for their own advantage. In this study, we investigated the association between murine norovirus (MNV) infection and the ISR and demonstrate that MNV regulates the ISR by activating and recruiting key ISR host factors. We observed that during MNV infection, there is a progressive increase in phosphorylated eukaryotic initiation factor 2α (p-eIF2α), resulting in the suppression of host translation, and yet MNV translation still progresses under these conditions. Interestingly, the shutoff of host translation also impacts the translation of key signaling cytokines such as beta interferon, interleukin-6, and tumor necrosis factor alpha. Our subsequent analyses revealed that the phosphorylation of eIF2α was mediated via protein kinase R (PKR), but further investigation revealed that PKR activation, phosphorylation of eIF2α, and translational arrest were uncoupled during infection. We further observed that stress granules (SGs) are not induced during MNV infection and that MNV can restrict SG nucleation and formation. We observed that MNV recruited the key SG nucleating protein G3BP1 to its replication sites and intriguingly the silencing of G3BP1 negatively impacts MNV replication. Thus, it appears that MNV utilizes G3BP1 to enhance replication but equally to prevent SG formation, suggesting an anti-MNV property of SGs. Overall, this study highlights MNV manipulation of SGs, PKR, and translational control to regulate cytokine translation and to promote viral replication.

## Abstract (precis)

Viruses hijack host machinery and regulate cellular homeostasis to actively replicate their genome, propagate, and cause disease. In retaliation, cells possess various defense mechanisms to detect, destroy, and clear infecting viruses, as well as signal to neighboring cells to inform them of the imminent threat. In this study, we demonstrate that the murine norovirus (MNV) infection stalls host protein translation and the production of antiviral and proinflammatory cytokines. However, virus replication and protein translation still ensue. We show that MNV further prevents the formation of cytoplasmic RNA granules, called stress granules (SGs), by recruiting the key host protein G3BP1 to the MNV replication complex, a recruitment that is crucial to establishing and maintaining virus replication. Thus, MNV promotes immune evasion of the virus by altering protein translation. Together, this evasion strategy delays innate immune responses to MNV infection and accelerates disease onset.

## INTRODUCTION

Human noroviruses (HuNoVs) are positive sense single-stranded RNA viruses that belong to the *Caliciviridae* family. They are a major cause of acute gastroenteritis in developing and developed countries (1–3). The onset of symptoms such as diarrhea, nausea, vomiting, and abdominal cramps usually commences 12 to 48 h after exposure to the virus and typically lasts no more than 48 h (4–6). Despite its significant health burden, there are currently no effective treatments or preventative vaccines for HuNoV infections, even though vaccines are under development (7–11). Advances in the use of antiviral agents to control HuNoV outbreaks have been severely delayed by the fact that HuNoVs are difficult to cultivate in the laboratory. Recent studies have shown that HuNoV is able to replicate in B-cell like cell lines when cocultured with specific enteric bacteria or in enteric organoids (12, 13). However, viral replication is poor with only a 2- to 3-log increase in viral titer, and thus the closely related genogroup V murine norovirus (MNV) remains a robust tissue culture system and small animal model (14).

The MNV genome is an ∼7.5-kb positive-sense RNA molecule that encodes 9 or 10 proteins (depending on translation of open reading frames [ORFs] and cleavage of gene products [15, 16]) that have roles in replication of the viral genome, polyprotein cleavage, translation, host manipulation, and assembly of virus particles. The 5′ end of the genome is covalently attached to viral protein g (VPg or NS5) and is polyadenylated at the 3′ end. The VPg protein mediates translation of the viral genome via interaction with host translation factors (17, 18). The remaining nonstructural proteins (ORF1) associate with the viral replication complex (RC) in induced membrane clusters (19, 20), as well as interacting with host factors to manipulate cellular homeostasis and promote viral replication. Not all proteins encoded by ORF1 have been functionally characterized, but previous studies revealed that the MNV NS1/2 protein associates with the endoplasmic reticulum (ER) and the host protein VAP-A (21, 22), whereas NS3 associates with microtubules and lipid-rich bodies in the cytoplasm (23). Further, NS7 acts as the RdRp (24, 25), and NS6 is the protease cleaving the polyprotein (26, 27).

Noroviruses cause acute and chronic infections that often involve manipulation of host processes and innate immune responses at multiple levels (reviewed in reference 28). The presence of viral double-stranded RNA (dsRNA) and proteins during MNV infection is recognized as foreign by the integrated stress response (ISR), and this can activate antiviral innate immune pathways. This recognition of infection can result in a myriad of responses, the most important being the type I and type III interferon (IFN) responses (15); however, the exact mechanisms employed to restrict and clear norovirus infections are not completely defined.

In the presence of cellular stressors, the ISR can be activated by eukaryotic initiation factor 2α (eIF2α) kinases such as dsRNA sensor protein kinase R (PKR), the ER-stress sensor PKR-like endoplasmic reticulum kinase (PERK), general control nonderepressible 2 kinase (GCN2), and heme-regulated kinase (HRI). The activation of these sensors can lead to the phosphorylation of eIF2α, which relinquishes eIF2α’s ability to bind to the 40S ribosomal subunit, prompting translational stalling and the aggregation of stalled translation preinitiation complexes (29–31). Together with the Ras-GAP SH3 domain binding protein (G3BP), T-cell restricted intracellular antigen 1 (TIA-1), and TIA-1-related protein (TIAR), these aggregates form stress granules (SGs) to stall translation and protect the cell from accumulating misfolded proteins during viral infections. SGs contain the preinitiation complexes that are typically comprised of various initiation factors, including eIF2, eIF3, eIF4α, eIF4β, eIF4G, and eIF5, as well as the 40S ribosomal subunit, which ensures that SGs reactivate translation rapidly after a successful stress recovery (32). SGs have also been shown to regulate and control cytokine mRNA aggregation and expression.

Several viruses manipulate the ISR to avoid immune detection by inhibiting translation and preventing the formation of SGs. Sindbis virus strongly inhibits the translation of cellular mRNA in PKR-dependent, as well as PKR-independent, mechanisms (33). Influenza A virus inhibits the phosphorylation of eIF2α and therefore prevents the induction of stress granules (34). Poliovirus, herpes simplex virus 1, and West Nile virus interferes with SG formation by cleaving or sequestering SG nucleating proteins such as G3BP and TIA-1 (35–37).

In this study, we demonstrate that MNV infection leads to the phosphorylation of eIF2α, via PKR, and the subsequent host cell translational shutoff, but this does not affect viral translation. However, restoration of active eIF2α does not alleviate host cell translational repression, suggesting that these events are uncoupled. Further, we show that this translational shutoff is associated with a decrease in cytokine translation and that MNV inhibits the formation of SGs by recruiting the SG nucleating factor G3BP1 to the sites of virus replication, a recruitment that is essential for MNV replication. Thus, we provide evidence that MNV manipulates the PKR–p-eIF2α–SG axis to promote its own replication but equally as an immune evasion strategy.

## RESULTS

### MNV infection induces eIF2α phosphorylation.

**FIG 1:**
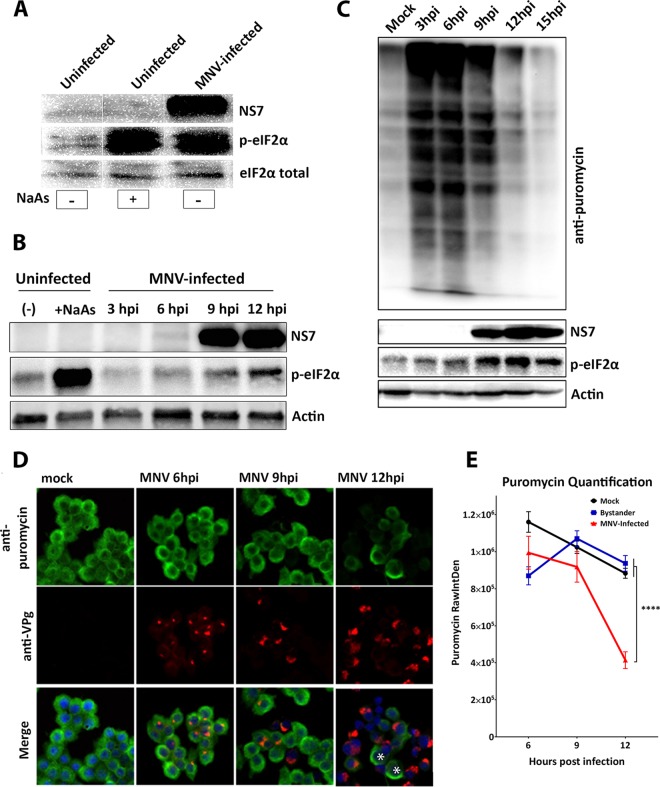
MNV infection phosphorylates eIF2α and shuts down host cell translation. (A) BMMs were uninfected, uninfected but NaAs treated (250 μM for 20 min), or MNV infected (MOI of 5) for 12 h. The WB was immunolabeled with anti-NS7, anti-p-eIF2α, and anti-eIF2α antibodies. Nonspecific lanes were removed to generate the image. (B) Immunoblot analysis of uninfected, uninfected/NaAs-treated (250 μM for 20 min), or MNV-infected (MOI of 5) cell lysates harvested at 3, 6, 9, and 12 hpi. The WB was immunolabeled with anti-NS7, anti-p-eIF2α and anti-actin antibodies. (C and D) BMM cells were either infected with MNV (MOI of 5) or left uninfected and analyzed for their translation using puromycin (10 μg/ml). (C) Immunoblot analysis of puromycin-treated (20 min) cell lysates harvested at 3, 6, 9, 12, and 15 hpi. The WB was immunolabeled with anti-puromycin, anti-NS7, anti-p-eIF2α, and anti-actin antibodies. (D) IF analysis of puromycin-treated (10 μg/ml for 30 min) cells at 6, 9, and 12 h postinfection. Cells were stained with anti-puromycin, anti-NS5, and DAPI for the merged image. Asterisks indicate uninfected cells displaying a high signal for anti-puromycin. Samples were analyzed via the Zeiss LSM 710 confocal microscope and analyzed with ZEN software. (E) Quantification of puromycin raw integrated density (RawIntDen) level of mock-infected and MNV-infected cells at 6, 9, and 12 hpi. 6 hpi, mock, *n* = 113, bystander, *n* = 108, and MNV, *n* = 38; 9 hpi, mock, *n* = 125, bystander, *n* = 108, and MNV, *n* = 40; 12 hpi, mock, *n* = 260, bystander, *n* = 148, and MNV, *n* = 81. Means ± the SEM are shown, and an unpaired two-tailed *t* test was performed. ****, *P* < 0.0001.

During our investigations of the intracellular replication of MNV we noticed changes in the level of host cell protein translation. To interrogate the influence of MNV on translation, we investigated whether MNV infection and replication induced eIF2α phosphorylation (Fig. 1A and B). Bone marrow-derived macrophage (BMM) cells were left untreated (mock), treated with the oxidative stressor sodium arsenite (NaAs; 250 μM for 20 min), or infected with MNV for 12 h. Our Western blot (WB) analysis of whole-cell lysates revealed that MNV infection, similar to the NaAs positive control, induced an increase in phosphorylated eIF2α (p-eIF2α) levels, whereas the total levels of eIF2α remained constant (Fig. 1A).

To further these initial observations, we investigated the kinetics of eIF2α phosphorylation throughout the course of the viral infection. Thus, MNV-infected cell lysates were collected at 3, 6, 9, and 12 h postinfection (hpi) and WB analysis was performed with antibodies for p-eIF2α, MNV NS7, and actin (Fig. 1B). Interestingly, we did not observe a change in eIF2α phosphorylation levels at 3 hpi compared to uninfected and untreated cells; however, as infection progressed, there was a gradual and noticeable increase in eIF2α phosphorylation at 6, 9, and 12 hpi. This establishes that MNV infection induces p-eIF2α as viral replication proceeds (Fig. 1B).

### eIF2α phosphorylation status corresponds to a repression of host cell protein translation during MNV infection.

One of the main consequences of eIF2α phosphorylation is the global shutoff of host cell protein translation (38, 39). To determine the effects of increasing eIF2α phosphorylation levels on cellular translation, BMM cells were infected with MNV and, at the indicated times postinfection (3, 6, 9, 12, and 15 hpi), the cells were pulsed with puromycin for 20 min prior to whole-cell lysate collection (for WB) and cell fixation (for immunofluorescence [IF]) (Fig. 1C and D, respectively).

Puromycin incorporates into newly translated polypeptides and terminates the translation of the full-length protein. Thus, newly synthesized proteins can therefore be visualized using an anti-puromycin antibody and accurately represents translational activity (40). Our WB analysis revealed that there was an increase in the amount of puromycin incorporated in active protein translation up to 6 hpi (Fig. 1C). However, as the infection progressed from 9 hpi (indicative by NS7 labeling) and the level of p-eIF2α increased, the levels of puromycin-labeled proteins were reduced (Fig. 1C). The WB results were supported by IF analysis demonstrating that incorporation of puromycin (Fig. 1D, green) begins to diminish in MNV-infected cells from 9 hpi, and by 12 hpi we observed a significant reduction in puromycin incorporation (Fig. 1D and E). Together, these results confirm that the MNV-induced increase in eIF2α phosphorylation correlates with a decrease in host cell protein translation. Interestingly, MNV protein translation (as determined by NS7 expression) steadily increases over the course of the infection even in the presence of eIF2α phosphorylation and host cell protein translation shut down (Fig. 1C).

### PKR induces the phosphorylation of eIF2α during MNV infection, but translation repression is PKR independent.

**FIG 2:**
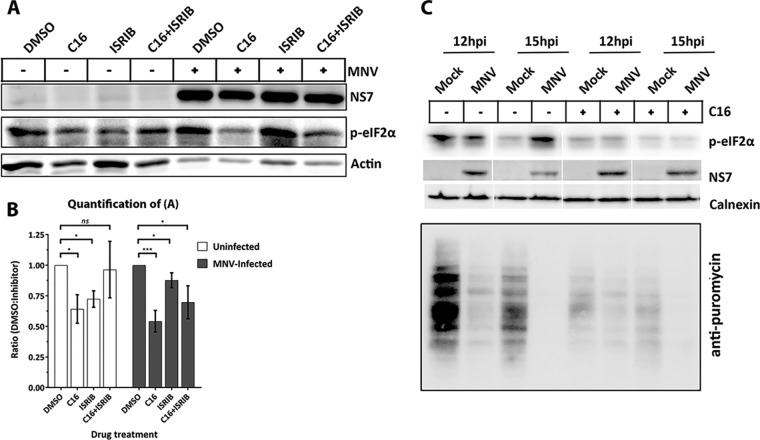
Treatment with PKR inhibitor C16 abolishes phosphorylation of eIF2α but does not rescue host translation. (A) RAW 264.7 cells were either uninfected or MNV infected (MOI of 5), treated with either DMSO, C16 (1 μM), ISRIB (0.5 μM), or C16+ISRIB at 1 hpi for 12 h before cell lysate samples were obtained. Lysates were analyzed via immunoblotting and immunolabeled with anti-NS7, anti-p-eIF2α, or anti-actin antibodies. (B) Quantification of p-eIF2α. Ratios: uninfected DMSO/C16 (1:0.64 ± 0.12, *P* = 0.015, *n* = 5), DMSO/ISRIB (1:0.72 ± 0.07, *P* = 0.0013, *n* = 3), and DMSO/C16+ISRIB (1:0.96 ± 0.23, *P* = 0.83, *n* = 3); MNV-infected DMSO/C16 (0.54 ± 0.09, *P* = 0.0008, *n* = 5), DMSO/ISRIB (0.88 ± 0.06, *P* = 0.03, *n* = 3, and DMSO/C16+ISRIB (1:0.70 ± 0.13, *P* = 0.02, *n* = 3). Statistics were calculated by using a Student *t* test; the means ± the SEM are shown. (C) RAW 264.7 were either uninfected or MNV infected (MOI of 5) and treated with either DMSO or C16 (1 μM) at 1 hpi for 12 or 15 h. At 30 min before the cell lysate samples were obtained, the cells were treated with puromycin (10 μg/ml for 30 min) and immunolabeled with anti-NS7, anti-p-eIF2α, anti-puromycin, and anti-calnexin antibodies.

During viral infection, PKR and PERK are two major kinases induced to prevent viral replication by phosphorylating eIF2α and inhibiting translation (41, 42). To investigate the potential role of PKR and/or PERK in mediating phosphorylation of eIF2α during MNV infection, we utilized a PKR inhibitor (C16), shown to suppress PKR-mediated phosphorylation of eIF2α (43), and a PERK inhibitor (ISRIB), shown to suppress PERK-mediated phosphorylation of eIF2α (44) to treat MNV-infected RAW 264.7 cells (Fig. 2A). Cells were infected with MNV and subsequently treated with 1 μM C16 and/or 0.5 μM ISRIB at 1 hpi. Cell lysates were collected at 12 hpi, and WB analysis was performed using an anti-p-eIF2α antibody. C16 treatment of MNV-infected cells substantially decreased the levels of p-eIF2α compared to untreated MNV-infected cells (Fig. 2B, dimethyl sulfoxide [DMSO]/C16 ratio, 1:0.54). In contrast, we observed a slight decrease in the p-eIF2α levels in the ISRIB-treated MNV-infected cells (Fig. 2B, DMSO/ISRIB ratio, 1:0.88). These results indicate that the phosphorylation of eIF2α observed during MNV infection is primarily mediated via PKR and not PERK.

Due to our observations that eIF2α phosphorylation was mediated via PKR, we speculated that inhibition of PKR activity would restore the repression of host cell protein translation. After MNV infection and C16 treatment, cells were incubated with puromycin before harvesting lysates at 12 or 15 hpi. Similar to previous results, protein translation is severely inhibited in the untreated MNV-infected cells; however, surprisingly, this phenotype was also maintained even in the presence of C16 and the lack of p-eIF2α (Fig. 2C). Thus, our results indicate that MNV-induced repression of host translation is uncoupled and independent of a PKR and p-eIF2α-mediated mechanism and must occur via a different regulatory pathway.

### The MNV NS3 protein induces host cell protein translation arrest.

Previous studies have suggested that the MNV protease NS6 can influence host cell protein translation via cleavage of the translation accessory factor PABP (45). To verify these observations, we expressed PABP-GFP in RAW 264.7 and infected the cells with MNV for 12 h. Neither the viral protein NS5 (VPg) nor NS6 (protease) colocalized with PABP-GFP in infected cells, and PABP-green fluorescent protein (GFP) expression was still observed (see Fig. S1A in the supplemental material). Further, we cotransfected cDNA expression plasmids encoding MNV NS3, NS6, or NS7 (RdRp) with PABP-GFP in HEK 293T cells. Upon cotransfection with NS6 and NS7, the PABP-GFP expression levels seemed unperturbed. However, cotransfecting PABP-GFP with NS3 significantly reduced PABP-GFP levels compared to the control (Fig. S1B). It is important to note that we did not detect any smaller sized protein bands for PABP-GFP that would indicate virus-induced cleavage of this protein, even in the presence of the MNV NS6 protease expressed during replication (Fig. S1B).

10.1128/mBio.00960-19.1 FIG S1 Host translation protein PABP is not cleaved during infection with MNV. (A) RAW 264.7 cells were transfected with a cDNA expression plasmid encoding PABP-GFP for 12hrs and then subsequently infected with MNV (MOI of 5) for an additional 12 h. Cells were fixed, permeabilized, and stained with antibodies against MNV NS5 (red), MNV NS6 (blue), and PABP-GFP is visualized in green. Samples were analyzed via confocal microscopy on a Zeiss 710 and images collated in Adobe Photoshop. (B) HEK 293T cell were transfected with cDNA expression plasmids encoding the MNV NS3, NS6, or NS7 proteins tagged with 6×His epitope and PABP-GFP for 18 h. Cell lysates were subsequently collected and analyzed via immunoblotting, and proteins were visualized with anti-6×His and anti-GFP antibodies. GAPDH was visualized with anti-GAPDH and utilized as a loading control. Download FIG S1, TIF file, 1.0 MB. © Crown copyright 2019. 2019 Crown This content is distributed under the terms of the Creative Commons Attribution 4.0 International license.

**FIG 3:**
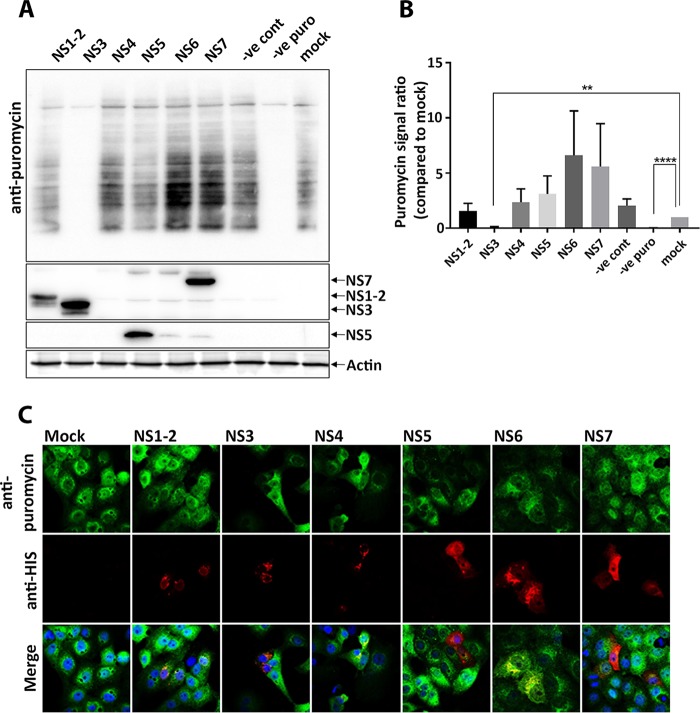
Expression of MNV NS3 protein abolishes host cellular translation. (A) HEK 293T cells were transfected with cDNA expression plasmids encoding the individual His-tagged MNV NS proteins for 18 h, at which point the cells were pulsed with puromycin as previously described, and whole-cell lysates were obtained and immunolabeled with anti-puromycin antibodies. (B) Densitometry analysis of puromycin signal in MNV NS protein-transfected cells compared to mock-transfected cells (*n* = 3, ANOVA, means ± the SEM; **, *P* < 0.01; ****, *P* < 0.0001). (C) Vero cells were transfected with single His-tagged MNV NS proteins and treated with puromycin before the cells were fixed and permeabilized for IF. Cells were immunolabeled with antibodies against puromycin (green), 6×His (red), and DAPI. Samples were captured by using a Zeiss LSM 710 confocal microscope and analyzed with ZEN software.

To determine which MNV proteins might affect host cell translation, we utilized puromycin incorporation in individual ORF1 protein-transfected cells. Thus, HEK 293T and Vero cells were transfected with plasmids encoding the single ORF1 proteins and treated with puromycin prior to immunoblot or IF analysis. We observed no significant change in the amount of incorporated puromycin in cells expressing MNV NS1/2, NS4, NS5, NS6, or NS7. Intriguingly, we observed a profound absence of puromycin incorporation in cells expressing the MNV NS3 protein (Fig. 3). These results suggest that the MNV NS3 has an impact on the host protein translational efficiency. In contrast to previous reports, we observed that the MNV NS6 protease did not influence host protein translation or cleave the translation accessory factor PABP (Fig. S1).

### MNV-induced suppression of host cell protein translation results in the inability of infected cells to produce major cytokines.

**FIG 4:**
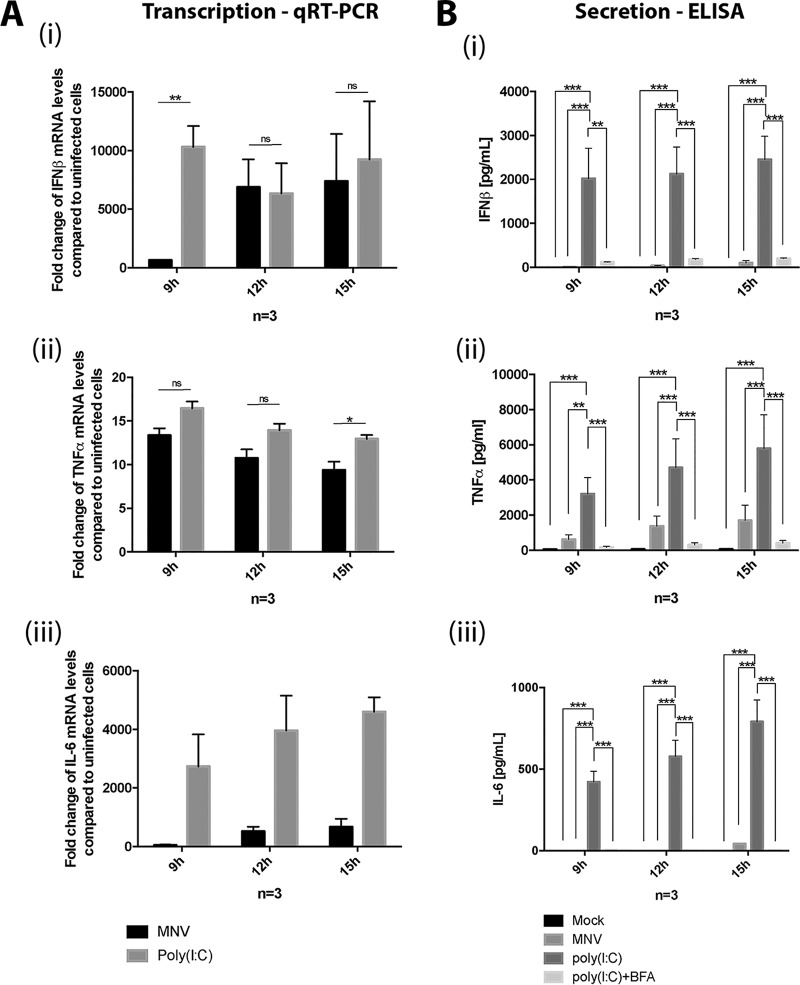
MNV infection in macrophages induces cytokine transcription but inhibits their secretion. (A) RAW 264.7 cells were either MNV infected (MOI of 5) for 9, 12, and 15 h or poly(I⋅C) treated. RNA samples were taken and analyzed via RT-qPCR for the cytokines IFN-β (i), TNF-α (ii), and IL-6 (iii). (B) RAW 264.7 cells were mock infected, infected with MNV (MOI of 5) for 9, 12, and 15 h, or poly(I⋅C) or poly(I⋅C)+BFA treated. Cell culture supernatants were analyzed for the secretion of the cytokines IFN-β (i), TNF-α (ii), and IL-6 (iii) by using ELISA (*n* = 3, ANOVA, means ± the SEM; **, *P* < 0.01; ****, *P* < 0.0001).

The innate immune response is crucial during MNV infection; specifically, STAT1 and type I and III interferons (IFNs) play essential roles in combatting infection (14). We were interested in investigating the impact of impaired protein translation on the innate immune response to MNV infection, and investigated the cytokines IFN-β, tumor necrosis factor alpha (TNF-α), and interleukin-6 (IL-6) since they represent major immune response pathways. First, we tested whether MNV infection induces the transcriptional activation of IFN-β, TNF-α, and IL-6. We infected RAW 264.7 with MNV, treated them with poly(I⋅C), or left them untreated for 9, 12, and 15 h. Transcription levels were assessed using reverse transcription-quantitative PCR (RT-qPCR) and compared to mock-treated cells (Fig. 4A). Poly(I⋅C) stimulation led to the robust induction of IFN-β, TNF-α, and IL-6 transcription, observed through increasing mRNA levels compared to untreated cells. MNV-infected cells also showed similar increases in mRNA levels for both IFN-β and TNF-α compared to poly(I⋅C)-treated cells (Fig. 4Ai and ii). However, although we observed a slight increase in IL-6 transcriptional response, this increase was much less profound (Fig. 4Aiii).

To test whether the translation of these major cytokines is affected by the global host translation shutoff during MNV infection, we infected RAW 264.7 cells with MNV, treated them with poly(I⋅C) and the secretion inhibitor brefeldin A (BFA), or left them untreated (Fig. 4B). Cell culture supernatant samples were harvested at 9, 12, and 15 hpi and cytokine secretion was measured via enzyme-linked immunosorbent assay (ELISA). Untreated cells, as well as cells stimulated with poly(I⋅C) but treated with BFA, secreted no or only small amounts of IFN-β, TNF-α, and IL-6 at any time point tested. In contrast, poly(I⋅C)-stimulated cells released large amounts of all three cytokines into the tissue culture supernatant as early as 9 h posttreatment. Interestingly, cells infected with MNV showed significantly smaller amounts of IFN-β, TNF-α, and IL-6 being secreted into the cell supernatant compared to poly(I⋅C)-treated cells (Fig. 4B). Cytokine levels observed for MNV-infected cells were similar to poly(I⋅C)- and BFA-treated cells, suggesting that secretion might be inhibited, comparable to the function of BFA. Surprisingly, general protein secretion is not disturbed in MNV-infected macrophages (Fig. S2), indicating that the reduction in protein levels is likely related to our observed MNV-induced translational inhibition.

10.1128/mBio.00960-19.2 FIG S2 MNV does not affect general protein secretion. (A) RAW 264.7 macrophages were transfected with the *Metridia* luciferase containing pBI-CMV5-mCherry vector. mCherry-positive cells were sorted and infected with MNV, treated with BFA, or left untreated. The relative luciferase activity was measured at 12 hpi (*n* = 3; average ± the SEM; ns, *P* > 0.05; *, *P* < 0.05; **, *P* < 0.01). (B) HEK 293T cells were transfected with pBI-CMV5 vectors containing the individual MNV NS proteins. As controls, pBI-CMV5 only and pBI-CMV5- and BFA-treated cells were used. Supernatants and lysates were collected at 24 h posttransfection, and the ratio between intracellular (lysate) and secreted (supernatant) luciferase activity was calculated (*n* = 3, average ± the SEM; ****, *P* < 0.0001). Download FIG S2, TIF file, 0.6 MB. © Crown copyright 2019. 2019 Crown This content is distributed under the terms of the Creative Commons Attribution 4.0 International license.

### MNV infection inhibits stress granule formation.

**FIG 5:**
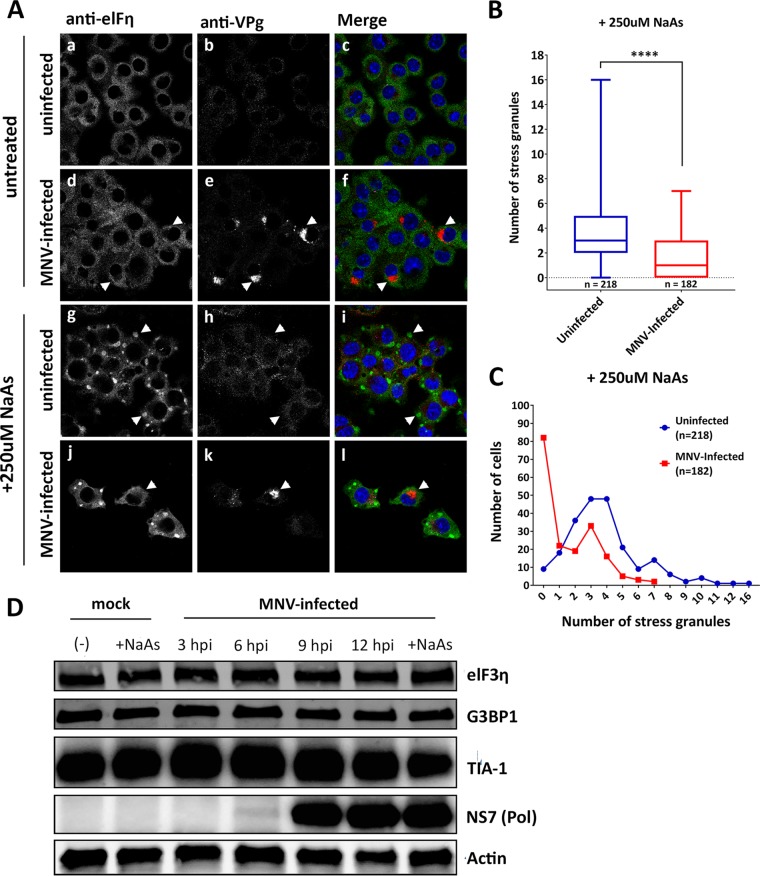
MNV infection alters SG formation in sodium arsenite-treated cells without affecting key SG protein levels. (A) BMMs were mock infected (a to c), infected with MNV (MOI of 5) for 12 h (d to f), NaAs treated (250 μM for 20 min) (g to i), or MNV infected and NaAs treated (j to l). The cells were fixed for IF analysis and immunolabeled with eIF3η (green), VPg (red, indicating infection), and DAPI (blue). Samples were captured via the Zeiss LSM 710 confocal microscope and analyzed with ZEN software. (B and C) SGs in NaAs-treated mock-infected (218 cells) and MNV-infected (182 cells) samples were counted from two independent experiments each and collated. (B) Box-and-whiskers plot, where the whiskers represent minimum to maximum, and the box represents the mean with error bars ± the SEM. An unpaired two-tailed *t* test was performed (****, *P* < 0.0001). (C) Quantitation demonstrating the total number of cells (*y* axis) containing various amount of SGs (*x* axis). The blue line represents the number of SGs in uninfected cells (*n* = 218 cells), and the red line represents the number of SGs in MNV-infected cells (*n* = 182 cells). (D) BMM cells were either mock infected or MNV infected (MOI of 5), and whole-cell lysates were collected at 3, 6, 9, and 12 hpi. A Western blot was immunolabeled with anti-eIF3η, anti-G3BP1, anti-TIA-1, anti-NS7, and anti-actin antibodies.

One of the control mechanisms for translation of IFN-stimulated genes and cytokines is the sequestering of the encoding mRNA within cytoplasmic RNA granules, e.g., SGs (46, 47). Based on our observed profound effect of MNV infection on host cell translation and innate immune-associated pathways, we aimed to investigate whether MNV replication also manipulated the formation of SGs. Thus, BMM cells were infected with MNV for 12 h, and cells were analyzed by IF with specific antibodies against the SG marker eIF3η and the viral VPg protein NS5 (Fig. 5A). In uninfected control cells, SG formation was not observed (Fig. 5A to C); however, treatment with NaAs induced the formation of numerous and obvious round cytoplasmic puncta (Fig. 5A, g to i, arrowheads). Interestingly, cells infected with MNV did not appear to contain SGs (Fig. 5A, d to f, arrowheads), suggesting that MNV infection either does not induce SG formation or that the virus inhibits the formation of SGs.

To determine whether MNV interferes with SG formation, cells were infected with MNV for 12 h and subsequently treated with NaAs (Fig. 5A, j to l). We observed an inhibitory effect of MNV on the amount of NaAs-induced SGs (Fig. 5A, j to l) compared to NaAs-treated uninfected cells (Fig. 5A, g to i). In NaAs-treated and MNV-infected cells exhibiting SG formation, the morphology of the SGs was smaller and elongated, instead of having a typical, round appearance (Fig. 5A, j to l). To quantitate the changes observed, we determined the number of SG foci within MNV-infected and uninfected cells in the presence of NaAs (Fig. 5B and C). MNV-infected cells displayed a significantly lower number of SGs within the cell compared to uninfected cells during NaAs treatment. In uninfected cells, an average of four SGs per cell were observed (Fig. 5B, blue), with only 4% of cells (9 cells) containing no SGs (Fig. 5C, blue). In contrast, infected cells had only an average of two SGs per cell (Fig. 5B, red), and 45% of infected cells (82 cells) contained no SGs at all (Fig. 5C, red). Intriguingly, our observations are in contrast to those of Humoud et al., who observed that MNV infection did not impact on arsenite-induced SG assembly (48).

Previous reports have indicated that some viruses prevent the induction of SGs by viral protease-mediated cleavage of G3BP1 and other accessory proteins (49). To determine whether this was also true for MNV infection, we investigated the protein levels of eIF3, G3BP1, and TIA-1 by WB. We observed no significant change in the total protein levels or size of any of these proteins as infection progressed, indicating that MNV does not manipulate SG formation through protease-mediated cleavage of key SG proteins (Fig. 5D). These results suggest that MNV does not induce SGs even though eIF2α is phosphorylated and has an inhibitory effect on SG induction.

### MNV recruits the key SG nucleating proteins G3BP1 to the sites of virus replication, which is critical for efficient MNV replication.

**FIG 6:**
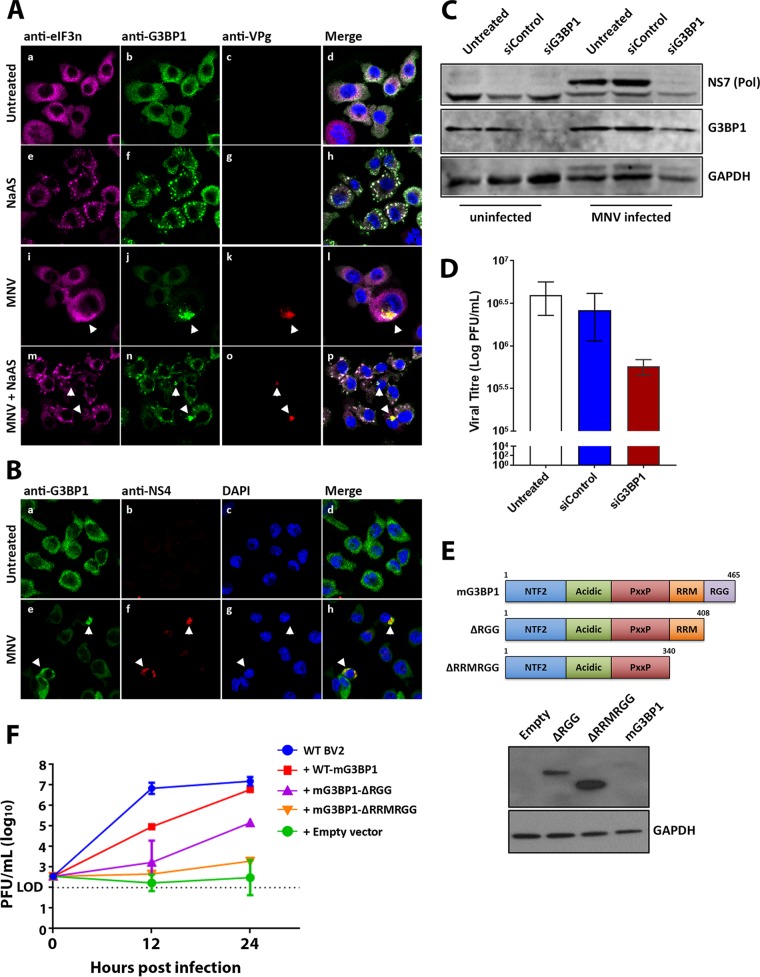
MNV recruits G3BP1 and requires G3BP1 for efficient viral replication. (A) BMMs were mock infected (a to d), NaAs treated (250 μM for 20 min) (e to h), infected with MNV (MOI of 5) for 12 h (i to l), or MNV infected and NaAs treated (m to p). Cells were fixed for IF analysis and immunolabeled with anti-eIF3η (magenta), anti-G3BP1 (green), VPg (red, indicating infection), and counterstained with DAPI (blue). (B) BMM cells were mock infected (a to d) or MNV infected (MOI of 5, 12 h) (e to h). Cells were fixed for IF analysis and immunolabeled with anti-G3BP1 (green) or anti-NS4 (red) and counterstained with DAPI (blue). (A and B) Samples were captured via the Zeiss LSM 710 confocal microscope and analyzed with ZEN software. (C and D) BMMs were untreated, siControl or siG3BP1 treated, and either mock infected or MNV infected (MOI of 5). (C) Whole-cell lysates were collected for WB (immunolabeled with anti-NS7, anti-G3BP1, and anti-GAPDH). (D) Tissue culture fluids were collected for plaque assay. *n* = 3 (means ± the SEM are shown, and an unpaired two-tailed *t* test was performed). (E) Schematic demonstrating WT-mG3BP1, mG3BP1-ΔRGG, and mG3BP1-ΔRRMRGG protein mutant constructs. Each construct was transfected into BV2, and the expression levels were demonstrated by WB. (F) WT-BV2 cells and BV2 -mG3BP1-KO cells were transfected with WT-mG3BP1, mG3BP1-ΔRGG, mG3BP1-ΔRRMRGG, and an empty vector. After transfection, the cells were infected with MNV, and at 12 and 24 hpi tissue culture fluids were collected for plaque assay. *n* = 3 (the means ± the SEM are shown, and an unpaired two-tailed *t* test was performed).

To examine the ability of MNV to prevent SG induction, we visualized the distribution of two key SG nucleating proteins, eIF3 and G3BP1, by IF analysis in MNV-infected BMM cells (Fig. 6). We observed no significant altered distribution of eIF3 in MNV-infected cells, either at the sites of viral replication or to discrete cytoplasmic foci (Fig. 6A, compare i to a). In contrast, we observed a dramatic redistribution and sequestering of G3BP1 to the sites of MNV replication as identified with antibodies to the MNV VPg protein (NS5) (Fig. 6A, compare j to b). The sequestering of G3BP1 also occurred in MNV-infected cells that were additionally treated with NaAs (Fig. 6A, compare n to f). To ensure that there was no unforeseen cross-reactivity between the anti-G3BP1and anti-VPg antibodies, we also performed IF analysis with anti-p20 (NS4) antibodies and observed the same result (Fig. 6B, compare h to d).

As we had observed that G3BP1 had been sequestered within the MNV RC, we sought to determine whether this was a functional consequence for evasion of the SG antiviral response or a requirement for replication. Intriguingly, in a recent CRISPR screen, G3BP1 was observed as the second most critical host factor, next to the receptor CD300lf, in facilitating MNV infection and replication (50). Since the CRISPR knockout of G3BP1 was observed to completely inhibit infection, we utilized RNAi-mediated suppression of G3BP1 to identify how MNV may require G3BP1 for replication. Thus, cells were incubated with different RNAi’s specific for the murine *G3bp1* gene, and the suppression of G3BP1 expression was assessed by WB analysis. Small interfering RNA (siRNA)-mediated treatment resulted in a reduction of the G3BP1 protein (Fig. 6C). Upon subsequent infection of these cells, we observed an attenuation in MNV replication by WB (NS7) (Fig. 6C) and the production of infectious virus by plaque assays (3.5/4-log reduction [representing an ∼80 to 90% decrease in infectious virus]) (Fig. 6D).

To further examine the impact of G3BP1 on MNV replication, we knocked out G3BP1 expression in BV2 cells via CRISPR-Cas9 (G3BP1-KO). In G3BP1-KO BV2 cells, we reintroduced G3BP1 by transfecting wild- type mouse G3BP1 (WT-mG3BP1) and two mG3BP1 protein deletion mutants: mG3BP1–ΔRGG (ΔRGG; deletion of the cooperative RNA binding domain [RGG] from amino acids 408 to 465 [aa408-465]) and mG3BP1–ΔRRMRGG (ΔRRMRGG; this also includes the RNA-binding domain [RRM] from aa340-407), as well as an empty vector (Fig. 6E and F). We infected these cells with MNV and collected virus containing tissue culture fluid at 12 and 24 hpi to measure viral titers. In WT-BV2 cells, peak virus replication (10^7^ PFU/ml) was observed after 12 hpi, and this level remained steady until 24 hpi (Fig. 6F, blue line). Consistent with our siRNA results, knockout of G3BP1 resulted in the complete abolishment of virus replication, highlighting the importance of G3BP1 for MNV replication (Fig. 6F, green line). Interestingly, the reintroduction of WT-mG3BP1 into mG3BP1-KO cells completely restored virus replication to WT BV2 levels (10^7^ PFU/ml) by 24 hpi; however, the rescue of MNV replication was delayed by 12 h (Fig. 6F, red line). Furthermore, the removal of the G3BP1–ΔRGG domain resulted in the partial rescue of MNV replication by 24 hpi (10^5^ PFU/ml, 2 logs lower than WT BV2) (Fig. 6F, purple line); however, the removal of the G3BP1–ΔRRMRGG domains resulted in a 4-log reduction of viral titers (Fig. 6F, orange line).

We suggest, based on these observations, that the recruitment of G3BP1 to the MNV RC is essential for virus replication. At this point we have not been able to identify at what stage and how G3BP1contributes to MNV replication; however, we speculate that it contributes to binding of the MNV viral RNA perhaps to stabilize some protein-RNA interactions. It is important to note that sequestration of G3BP1 is also critical to prevent SG formation and promote viral replication without interference of the innate immune response.

## DISCUSSION

The shutdown of host cell translation is one of the major host defense mechanisms against viral infections. Viral replication is completely dependent on host cell translation, since viruses lack their own translational machinery and parasitize the hosts. Therefore, a reduction in host protein translation will likely lead to a decreased translation of viral proteins and interfere with efficient viral replication. Interestingly, translation of viral proteins such as NS7, the viral polymerase, does not seem to be affected by the reduced host protein translation during MNV infection because intracellular amounts of NS7 increase from 6 hpi onward, whereas host protein translation subsides (Fig. 1B). These observations strongly suggest that MNV employs an alternative mechanism to initiate translation, independent of cellular protein translation (51). The HuNoV and MNV VPg proteins have been shown to interact with the translation initiation complex through eIF4GI and eIF4E, suggesting a role of NS5 in the initiation of viral protein translation (52, 53). Translation of MNV proteins, which is independent of the cellular cap-dependent protein translation, could be mediated by VPg and allow viral protein translation to occur in the absence of cellular protein translation (54). This would be a great advantage for the virus, not only by forcing the cell to preferentially translate viral proteins but also by diminishing the innate immune response by preventing the translation of immune effectors such as cytokines.

To uncover how MNV manipulates the ISR, we investigated the PKR/eIF2α pathway, which is a major regulator of the ISR (Fig. 1 to 3). We demonstrated that MNV infection leads to the phosphorylation of eIF2α, supporting the observations by Humoud et al. (48), and as infection progresses the amount of p-eIF2α drastically increases, resulting in timely host cell translational shutoff (Fig. 1). Even though translation was upregulated early during the infection (6 hpi), there was a continuous decrease in the amount of puromycylated proteins at later stages of the infection (from 9 hpi), indicating a reduction in global host cell translation (Fig. 1B). Based on immunoblot and IF analyses (Fig. 1C), MNV starts to affect host cell translation from 9 hpi, reducing host cell translation to a minimum in most infected cells by 12 hpi (Fig. 1C). Expression studies of single viral proteins revealed that NS3 expression alone is sufficient to induce translation inhibition (Fig. 3).

We presumed that p-eIF2α may be regulated via PKR which is activated by binding to dsRNA produced during MNV infection. We initially showed that phosphorylation of eIF2α was mediated via PKR rather than PERK (Fig. 2). Interestingly though, when cells were treated with C16 and analyzed for their translation activity via puromycin treatment, we did not observe an increase in host cell translation activity in MNV-infected cells compared to infected, but untreated cells (Fig. 2B). These observations show that the PKR-eIF2α axis is activated during MNV infection but is not solely responsible for the host cell translation shutdown. However, the shutdown of host translation by MNV is effective and robust (Fig. 3), and we show that it affects the translation of innate immune response regulators such as cytokines (Fig. 4).

The release of cytokines such as IFN-β, TNF-α, and IL-6 during viral infections plays a crucial role in the innate immune response against viruses. The transcription and translation of cytokines are elevated in virus-infected cells, mostly due to the recognition of pathogen-associated molecular patterns (PAMPs), e.g., dsRNA. Cytokines are then secreted into the extracellular space, where they can either bind to receptors on neighboring cells or to receptors on the infected cell itself to enhance the antiviral response (55). We and others have shown that MNV-infected cells increase the transcription of cytokine (IFN-β, TNF-α, and IL-6) mRNAs (Fig. 4) (56, 57), indicating that pattern recognition receptors (PRRs) such as MDA5 (58) have successfully detected the viral infection and activated an antiviral response against it. Intriguingly, MNV-infected cells do not secrete significant levels of cytokines which would help to overcome and contain the acute infection (Fig. 4). Our subsequent studies revealed that the small amounts of secreted cytokines from infected cells were not due to the inhibition of general protein secretion (Fig. S2). Instead, we observed that only very small amounts of translated cytokines can be detected within the infected cells, further confirming that there is no secretion inhibition, which would cause the accumulation of cytokines within the cells (Fig. 4). The difference in intracellular protein levels for TNF-α compared to the mRNA levels indicates an interference of the virus with either protein stability or the translation of host cell proteins.

Like all viruses, MNV must modulate host responses to provide conditions suitable for intracellular replication. To replicate successfully, MNV must also control the localization of viral RNA within the host cell, since these replication by-products are highly immunostimulatory. SG formation, which is part of the ISR, generates cytoplasmic granules containing stalled translational machineries involved in regulating RNA transcript homeostasis (59). This mechanism serves as an extension of translation by sequestering mRNA from active translation, while allowing the translation of certain mRNAs. This translational regulation is typically induced upon exposure to cellular stresses, including ER stress, oxidative stress, heat shock (60, 61), and viral infection (59). Under stressed conditions, cells activate eIF2α kinases to phosphorylate eIF2α, which depletes the eIF2α-GTP-tRNA^Met^ ternary complex required to form the preinitiation complex, resulting in stalled translation initiation (30, 31). These stalled preinitiation complexes aggregate and form SGs; in this way, general protein translation is inhibited (29). This host response to infection can affect cytokine translation; thus, some viruses have devised strategies to regulate RNA granule function to selectively control RNA translation and therefore promote their replication (reviewed in references 28 and 62).

Previous studies have shown that many different virus families modulate SG function to allow efficient replication (63). Thus, as we observed eIF2a phosphorylation in MNV-infected cells, we extended our IF analysis to examine whether SGs form during MNV infection. We demonstrated via IF that SGs are reduced during MNV infection, although eIF2α is phosphorylated. In fact, when cells are treated with NaAs, MNV infection significantly dampened SG formation and SGs that were present had atypical morphologies and reduced SG numbers (Fig. 5A and B). These results suggest not only that MNV infection does not induce SG formation but that MNV can also exert control over SG formation. Intriguingly, Humoud et al. (48) did not observe these findings, and it is difficult to reconcile their findings with ours. The only difference is that they utilized J774 macrophages in their study, potentially identifying subtle cell type differences.

We have shown that MNV recruits G3BP1 to sites of viral replication (Fig. 6A and B) and demonstrate through siRNA-mediated knockdown and CRISPR-Cas9 depletion of G3BP1 that it is required for efficient viral replication (Fig. 6C to F). We postulate that MNV recruits G3BP1 to sites of viral replication, where G3BP1 binds to viral RNA via the RNA recognizing motif (RRM). G3BP1 recruitment to the assembly complex seems to serve a dual purpose: (i) the promotion of viral replication (presumably by aiding in RNA duplex unwinding) and (ii) the prevention of SG formation.

**FIG 7:**
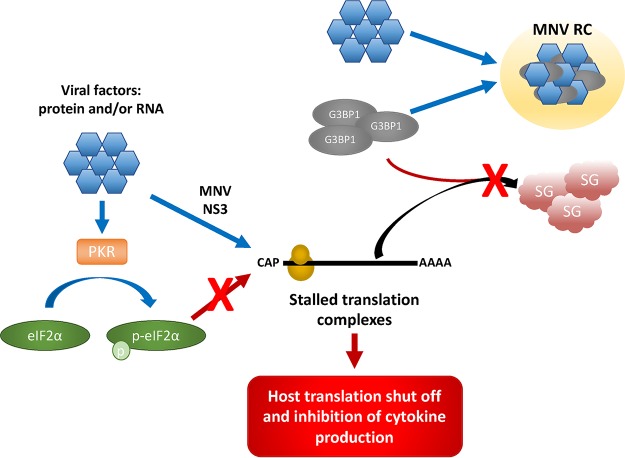
Model. During MNV infection, viral factors such as proteins and/or RNA (blue hexagon) phosphorylate eIF2α (green oval) via PKR (orange rectangle), as well as stalling translation initiation by the MNV NS3 protein. However, this translational arrest is uncoupled from the PKR–p-eIF2α axis. These stalled preinitiation complexes typically aggregate with G3BP1 (gray oval) and form SGs (red cloud). However, MNV viral factors sequester G3BP1 to the MNV RC (yellow circle) to promote replication. This allows the inhibition of cap-dependent host cell translation, as well as inhibiting the formation of SGs.

Based on our findings, MNV likely employs a strategy similar to picornaviruses and alphaviruses to evade the innate immune response by inducing the inhibition of host cell translation (64–67). During MNV infection, shutdown of host translation is independent of the SG-PKR-eIF2α axis and PABP cleavage and seems to be regulated through an unknown mechanism (Fig. 7, model). It will be interesting to investigate whether MNV cleaves other components of the translation complex, or whether it regulates the host translation shutdown through another pathway, such as miRNA or preferred binding of viral mRNA to the translation complex. Overall, it is important to note that the MNV NS3 protein allows the inhibition of cap-dependent host cell translation, while inhibiting the formation of SGs by recruiting G3BP1 to the MNV RC, which sequester stalled pretranslation complexes containing essential components of the translational machinery (Fig. 7, model). It is intriguing to speculate that MNV selectively induces cap-dependent translation inhibition to enhance viral translation and inhibit the innate immune response but needs access to the translational machinery and therefore inhibits SG formation. This strategy not only increases viral replication efficiency but also promotes immune evasion of the virus, which could explain the rapid replication cycle and the delayed innate immune response to MNV infection.

## MATERIALS AND METHODS

### Cell lines.

RAW 264.7 murine macrophages, bone marrow-derived macrophages (BMMs), and Vero and HEK 293T cells were maintained in Dulbecco modified Eagle medium (DMEM; Gibco) supplemented with 10% fetal calf serum (FCS; Gibco) and 1% GlutaMAX (200 mM; Gibco). BV2 cells were cultured in DMEM with 10% FBS, 1% HEPES, and 1% GlutaMAX. All cell lines were cultivated at 37°C in a 5% CO_2_ incubator, as previously described (19).

### MNV infection.

RAW 264.7 macrophages, BMMs and BV2 cells were infected with MNV at a multiplicity of infection (MOI) of 5, as previously described (19). Cells were rocked in a low volume of media for 1 h at 37°C, before the cells were supplemented with additional media. Unless indicated differently, cells were fixed or lysed at 12 hpi. If supernatant was collected, the medium was centrifuged at 10,000 × *g* for 3 min to pellet cellular debris.

### Lipofectamine 2000 DNA transfection.

Seeded cells were incubated until 80% confluence. First, 1 μg of DNA in 50 μl of Opti-MEM (Gibco) and 1.5 μl of Lipofectamine 2000 (Life Technologies) in 48.5 μl of Opti-MEM were incubated at room temperature for 2 min. The DNA and Lipofectamine mixtures were combined and incubated for a further 10 min. Meanwhile, the cells were washed with fresh cell culture media. Next, 400 μl of cell culture medium was added to each well. On the top, the DNA-Lipofectamine mixture was added dropwise. Cells were incubated at 37°C until required. The method used here was designed for a 24-well plate. The protocol was scaled according to the plate used.

### Chemicals, plasmids, and antibodies.

Sodium arsenite (Sigma-Aldrich) was added to the cells at a concentration of 250 μM for 20 min prior to fixation or cell lysate collection. The PKR inhibitor C16 (Sigma-Aldrich) was added to infected cells at a concentration of 1 μM at 1 hpi, and cell lysates were collected at 12 hpi. The ISR inhibitor ISRIB (Sigma-Aldrich) was added at 1 hpi at 0.5 μM. Puromycin (Life Technologies) was added to cells at a concentration of 10 μg/ml at indicated times prior to cell lysate collection. Poly(I⋅C) (Sigma-Aldrich, catalog no. P1530) is a dsRNA analogue and was used as a positive control for immune activation. Poly(I⋅C) was used at a concentration of 20 μg/ml and added to the cell culture medium in combination with Lipofectamine 2000. MNV ORF1 plasmids were described and used previously (22).

Goat anti-eIF3η, goat anti-G3BP1, and goat anti-TIA-1 were all purchased from Santa Cruz Biotech. Rabbit anti-eIF2α was purchased from Invitrogen; Rabbit anti-actin from Sigma-Aldrich; Mouse anti-puromycin was obtained from Kerafast, Inc. Mouse anti-G3BP1, mouse anti-GAPDH, rabbit anti-His, and rabbit anti-calnexin were obtained from Abcam, and rabbit anti-p-eIF2α (S52) and Alexa Fluor-conjugated species-specific IgG were purchased from Life Technologies. Rabbit anti-NS7 and rabbit anti-NS5 were manufactured and produced by Invitrogen.

### Plaque assay.

A total of 3.0 × 10^5^ RAW 264.7 or BV2 cells were seeded onto 12-well plates and incubated 37°C until 70% confluent. Virus-containing supernatants were 10-fold serially diluted in DMEM, added to plates, and rocked every 10 min for 1 h at 37°C. After incubation, a plaque assay overlay (70% DMEM, 2.5% [vol/vol] FCS, 13.3 mM NaHCO_3_, 22.4 mM HEPES, 200 mM GlutaMAX, and 0.35% [wt/vol] low-melting-point agarose) was added to each well. The overlay was solidified at 4°C for 15 min, followed by incubation at 37°C for 48 h. The cells were fixed in 10% formalin for 1 h at room temperature. The plaque assay overlay was removed, and the cells were stained with 1 ml of toluidine blue for 30 min. The stain was removed, the samples were rinsed with water, and plaque formations were enumerated.

### Immunofluorescence microscopy.

Cells were rinsed twice with phosphate-buffered saline (PBS) and fixed using 4% (vol/vol) paraformaldehyde/PBS for 15 min at room temperature. The fixative was removed, and the cells were permeabilized with 0.1% (vol/vol) Triton X-100 for 10 min at room temperature. The cells were rinsed twice with PBS and quenched with 0.2 M glycine for 10 min at room temperature. The cells were then rinsed with PBS, and coverslips were incubated in primary antibodies diluted in 25 μl of 1% bovine serum albumin (BSA)/PBS for 1 h at room temperature. After incubation with primary antibodies, the cells were washed three times with 0.1% BSA/PBS. Coverslips were incubated in secondary antibodies diluted in 25 μl of 1% BSA/PBS for 45 min at room temperature. The cells were washed twice with PBS and incubated for 5 min with DAPI (4′,6′-diamidino-2-phenylindole at 0.33 μg/ml in PBS. The coverslips were rinsed twice with PBS and Milli-Q water and mounted on cover slides with ProLong Diamond (Life Technologies). The cells were analyzed using a Zeiss LSM710 confocal microscope.

### Western blot analysis.

Cells were lysed with NP-40 lysis buffer containing protease inhibitor cocktail and phosphatase inhibits. Samples were separated on a bis/tris polyacrylamide gel and transferred to a polyvinylidene difluoride membrane. The membrane was blocked with 5% BSA/PBS-T (PBS plus Tween) for 2 h. Primary antibodies were added in 5% BSA/PBS-T and incubated overnight at 4°C. The following day, the membrane was washed three times with PBS-T and incubated with secondary antibody in PBS-T for 90 min at room temperature (using the Invitrogen antibodies Dk Anti-Ms AF488 [catalog no. A21202], Dk Anti-Rb AF594 [catalog no. A21207], and Dk Anti-Ms AF647 [catalog no. A21447]). The membrane was then washed four times in PBS-T and visualized using the MF-ChemiBIS DNR (Bio-imaging Systems).

### ELISA.

Cell culture supernatants were analyzed for their cytokine concentrations using mouse-specific ELISA kits for the following cytokines: IFN-β (Legend Max; BioLegend) and TNF-α (ELISAkit). Tissue culture supernatants and standards were applied to the 96-well precoated assay plate and incubated for 2 h. Wells were washed four times in assay wash buffer before adding assay-specific biotin-labeled detection antibody. Plates were incubated for 2 h at room temperature, and the wells were washed four more times with assay wash buffer. A streptavidin-conjugated horseradish peroxidase was then added, followed by incubation for a further 45 min. The plates were washed thoroughly six times with wash buffer, and TMB substrate was added. Plates were checked every 3 to 5 min to observe the color change. The reaction was stopped with assay stop solution. The absorbances at 450 and 570 nm (background) were measured using a CLARIOstar microplate. Cytokine concentrations were calculated using the ELISAanalysis.com website.

### qRT-PCR.

Cells for RNA extraction were lysed with TRIzol (Life Technologies). The total RNA was isolated by phenol-chloroform extraction and then stored at –80°C. The total RNA concentration was quantified using a NanoDrop apparatus, and 1 μg of total RNA was treated with RQ1 DNase (Promega) at 37°C for 45 min. cDNA was generated by reverse transcription using Sensifast RT (Bioline) at 25°C for 10 min, 42°C for 15 min, and 85°C for 5 min. cDNA levels were quantified by qPCR with SYBR GreenER (Bio-Rad) under the following cycling conditions: 50°C for 8 min, 95°C for 2 min, 40 cycles of 15 s at 95°C, and 1 min of annealing/extension at 60°C, followed by a final extension for 10 min. The fold induction of RNA was compared to the housekeeping gene (GADPH), and error bars indicate means ± the standard errors of the mean (SEM) from triplicate experiments.

### RNAi-mediated depletion of G3BP1.

BMM cells were reverse transfected with 0.25 μM siRNA (Bioneer) and RNAiMAX (Invitrogen) in Opti-MEM (Gibco). Cells were incubated at 37°C and 5% CO_2_ for 24 h. The following day, the cells were once again transfected with 0.5 μM siRNA. At 24 h posttransfection, the cells were infected with MNV at an MOI of 5 for 1 h, transfected with 0.5 μM siRNA, and incubated for a further 12 h. At 12 h postinfection, whole-cell lysates and supernatants were collected for WB and plaque assay, respectively.

### Generation of G3BP1 KO cells via CRISPR-Cas9.

BV2 cells were cultured in DMEM containing 10% FBS and 1% HEPES. BV2 cells were transiently transfected with Cas9 and a sgRNA (5′-TTCCCCGGCCCCGGCTGATGNGG-3′) targeting exon 7 of G3BP1. BV2 cells were then single cell cloned, and G3BP1 was sequenced using Illumina HiSeq. BV2 cells are polyploid at the G3BP1 locus, as described previously (PMID 27540007). Clone 1A3 had four independent deletions at the sgRNA binding site resulting in two unique 1-bp deletions, in addition to 4- and 10-bp deletions. The mutations introduced resulted in frameshifts and the absence of detectable G3BP1 protein, as measured by WB. Sequences are available upon request.

10.1128/mBio.00960-19.3 TEXT S1 Supplemental methods and results. Download Text S1, DOCX file, 0.02 MB. © Crown copyright 2019. 2019 Crown This content is distributed under the terms of the Creative Commons Attribution 4.0 International license.
