# Supplementary figures and images for: Mouse Norovirus Infection Arrests Host Cell Translation Uncoupled from the Stress Granule-PKR-eIF2α Axis
Source: mBio. 2019 Jun 18;10(3):e00960-19. doi: 10.1128/mBio.00960-19 (PMC6581855; doi:10.1128/mBio.00960-19)

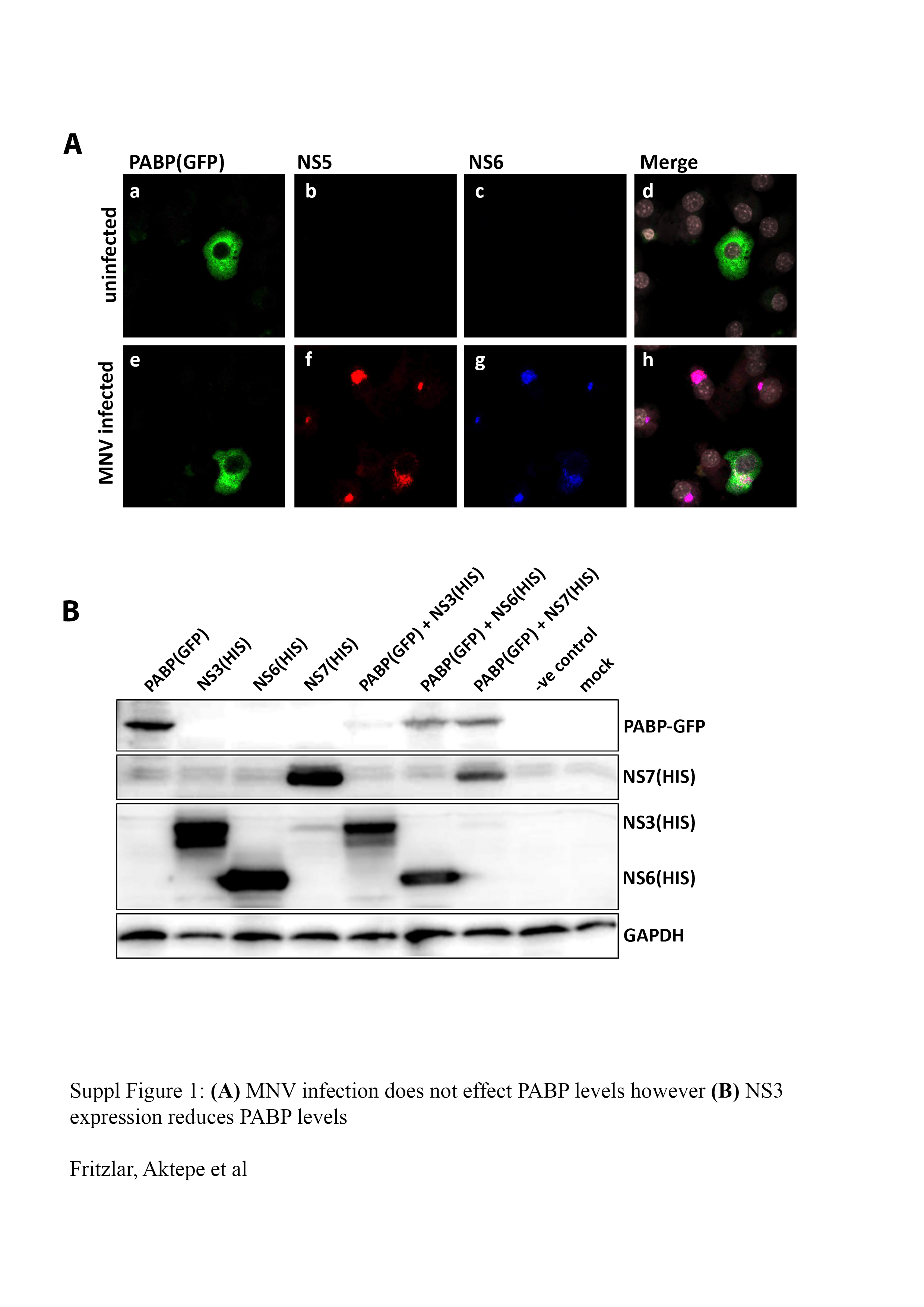

Supplement: FIG S1 [file mBio.00960-19-sf001.tif]

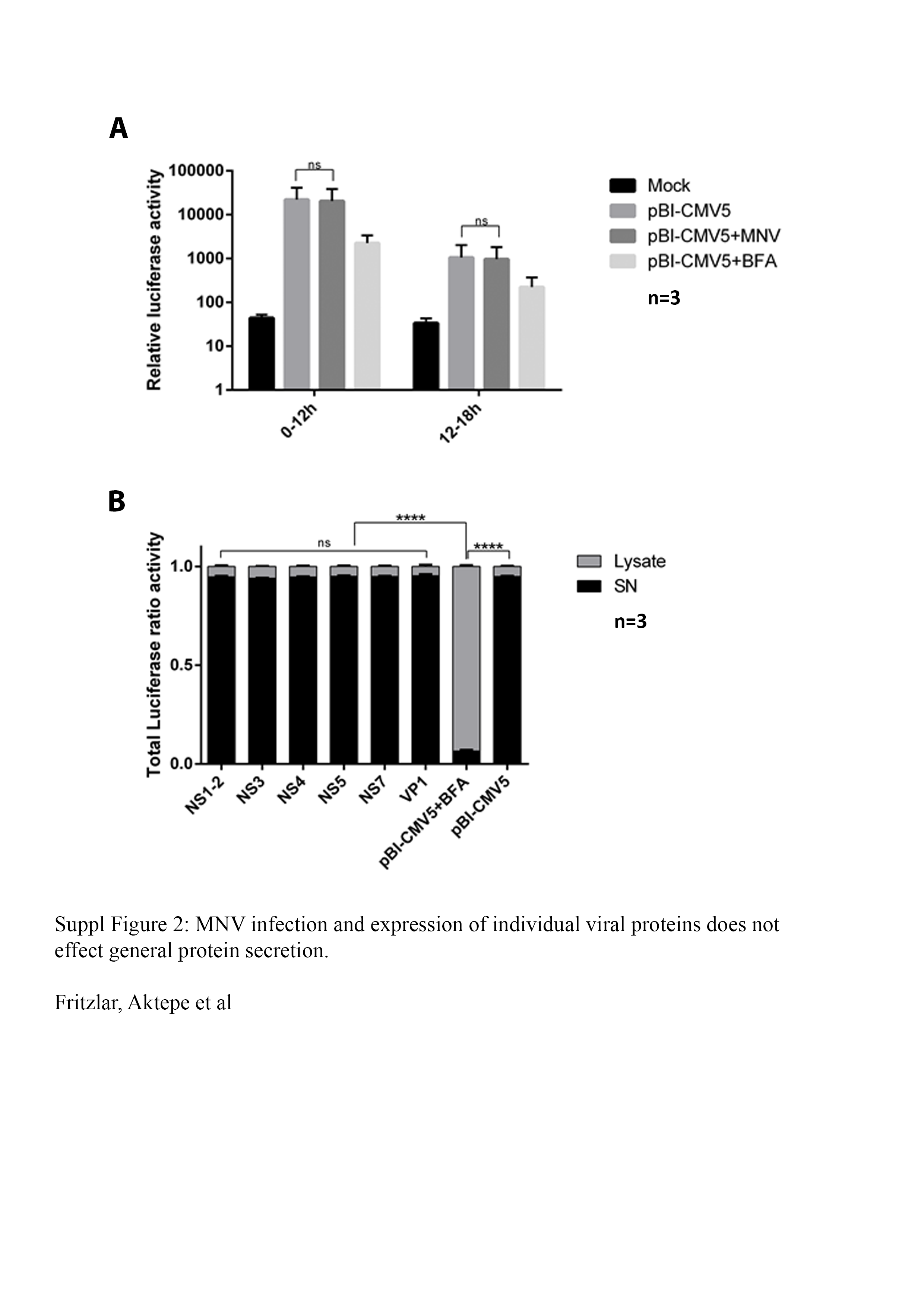

Supplement: FIG S2 [file mBio.00960-19-sf002.tif]
